# A unicentric center, multicenter, and mixed-type Castleman disease: Three case reports and a review of the literature

**DOI:** 10.1097/MD.0000000000037722

**Published:** 2024-04-12

**Authors:** Yang Xu, Shuai Shao, HaoNan Kang, ZhaoHui Xu, GuoYi Wen, Yan Shan, ZeZhong Gong, Abdulkarem Al-Sharabi, BoXin Qu, YanYing Ren, Fan Zhang, JinMing Guan, Xin Chen

**Affiliations:** a Department of Hernia and Colorectal Surgery, The Second Hospital of Dalian Medical University, Dalian, People’s Republic of China; b Department of Minimal Invasive Intervention Radiology, Ganzhou People’s Hospital, Ganzhou, People’s Republic of China.

**Keywords:** case report, Castleman disease, retroperitoneal disease

## Abstract

**Rationale:**

Due to the lack of specificity symptoms and site of onset of castleman disease (CD), it is difficult to diagnose and poses unique challenges for both patients and clinicians, leading to confusion in diagnosis and delays in treatment. To enhance understanding, we present 3 cases of CD treated at our hospital, including a single-center, multicenter, and mixed-type CD.

**Patient concerns:**

Case 1: A 53-year-old female patient was admitted with a chief complaint of “abdominal pain and fever for 10 days.” Marked enlargement of inguinal lymph nodes on both sides was observed. Case 2: A 58-year-old female patient was admitted with the main complaint of “discovering a left lower abdominal mass during a routine checkup for the past 10 days.” Upon deep palpation, a palpable mass of approximately 5.0 * 3.0 cm was identified in the left lower abdomen. Case 3: A 40-year-old male patient was admitted with the main complaint of “progressive right upper abdominal and lumbar back pain for over 6 months.” Computed tomography examination revealed multiple nodular soft tissue masses between the abdominal aorta and inferior vena cava, with the largest measuring 5.0 * 4.0 cm.

**Diagnoses:**

Based on the immunohistochemical results, the diagnoses for the 3 patients are as follows: Case 1: Multicentric Castleman’s Disease (Mixed Type). Case 2: Pelvic Retroperitoneal Castleman Disease (Hyaline Vascular Type). Case 3: Castleman Disease Multicentric Type.

**Intervention:**

Case 1: cyclophosphamide 0.6–1 g + vincristine 2 mg + methylprednisolone 50 mg/5 days. Cyclophosphamide 1 g + prednisone 30–50 mg/5 days. This alternating chemotherapy cycle is repeated every 6 months. Case 2: Laparoscopic pelvic mass excision surgery. Case 3: Surgical excision of the mass.

**Outcomes:**

Case 1: After a 43-month follow-up, the patient’s general symptoms have improved compared to before, but regular chemotherapy is still necessary at present. Case 2: The patient did not take any medication postoperatively, and there has been no evidence of metastasis or recurrence during the 18-month follow-up. Case 3: The patient did not take any medication, and there has been no evidence of metastasis or recurrence during the 21-month follow-up.

**Lessons subsections:**

The lack of specific signs on imaging studies and nonspecific blood tests increases the difficulty of diagnosis. However, tissue biopsy remains a feasible option. Therefore, we recommend conducting thorough examinations for suspected CD patients to reduce misdiagnosis and determine the CD type for effective targeted treatment.

## 1. Introduction

Castleman disease (CD), also known as angiofollicular lymph node hyperplasia, is a rare lymphoproliferative disorder. It can occur anywhere in the body, leading to involvement of corresponding organs. The disease presents in lymph nodes, and its manifestations resemble those of other benign and malignant conditions.^[1]^ It has a tendency to transform malignantly and is commonly found in the Mediterranean and African regions.^[2]^ The disease is rare in China, and due to the lack of specific symptoms and distinctive disease locations, some clinicians have limited awareness, leading to potential misdiagnosis or underdiagnosis.

Initially, CD presents without symptoms. Clinical manifestations only emerge when the affected lymph nodes enlarge to the extent of exerting pressure on nearby structures. As the disease progresses, patients exhibit a series of signs and symptoms, including multicentric lymph node involvement, multi-organ system dysfunction, inflammatory syndromes, and fluid accumulation.^[3,4]^ The true incidence of the disease is currently unclear. Literature suggests that risk factors for CD development include human immunodeficiency virus infection, male gender, inflammation, autoimmune conditions, and viral infection (Human Herpesvirus-8).^[5–7]^ Early diagnosis and treatment of this disease are crucial for improving the quality of life and increasing patient survival.

To enhance understanding, we present 3 cases of Castleman Disease treated at our hospital, including a single-center, multicenter, and mixed-type CD, as follows:

*Case 1*: A 53-year-old female patient was admitted with a chief complaint of “abdominal pain and fever for 10 days.” The patient had experienced continuous upper abdominal bloating and fever for the past 10 days without any apparent cause. There was no radiation of pain to the chest or back. The highest recorded body temperature was 38.9 °C, and the patient also had lower limb swelling. There were no chills or diarrhea, and the patient had regular gas and bowel movements. The patient had received treatment for acid suppression and fever reduction at a lower-level hospital, but her symptoms worsened. Therefore, further examinations were performed, and she was admitted to the emergency department with a provisional diagnosis of “pancreatitis and nephrotic syndrome.” Recently, the patient had poor dietary and sleep habits, and there was no significant medical history or family history.

Physical examination revealed distension of the entire abdomen, marked tenderness, rebound tenderness, and positive shifting dullness. There was significant enlargement of lymph nodes in both inguinal areas, and the lower limbs showed non-concave edema with asymmetric involvement.

Laboratory tests indicated red blood cells in urine at 5.6/HP, urinary protein (++), amylase at 260 U/L, plasma albumin at 22.7 g/L, platelets at 30 * 10^9^/L, hemoglobin at 78 g/L, urinary creatinine at 22 mmol/d, C-reactive protein at 100.62 mg/L, negative bacterial culture, negative tuberculosis nucleic acid test, negative viral tests, and negative malignancy markers.

**Figure 1.**
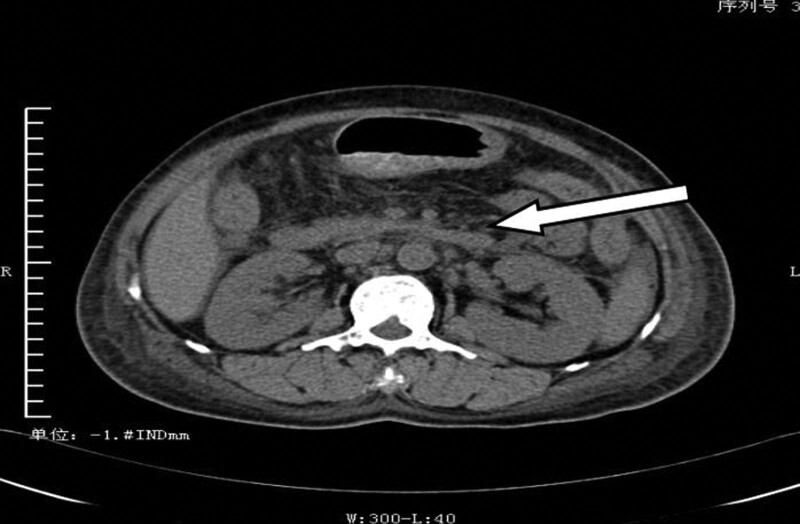
The abdominal CT scan reveals enlargement of the body skin, abdominal cavity, and pelvic lymph nodes. CT = computed tomography.

Imaging studies revealed enlarged lymph nodes throughout the body, abdominal and pelvic cavities (Fig. 1), pleural effusion, and pericardial effusion. Two bone marrow routine biopsies showed mild microcytic anemia.

Immunohistochemistry of the superficial lymph nodes showed CD3(+), CD5(+), CD20(+), CD21(FDC+), CD68(+), Kappa(+), Lambda(+), IgG(+), IgG4 (occasionally +), S-100 (scattered +), Ki67(30%+), HIC8(−), and EBER (occasionally +).

**Figure 2.**
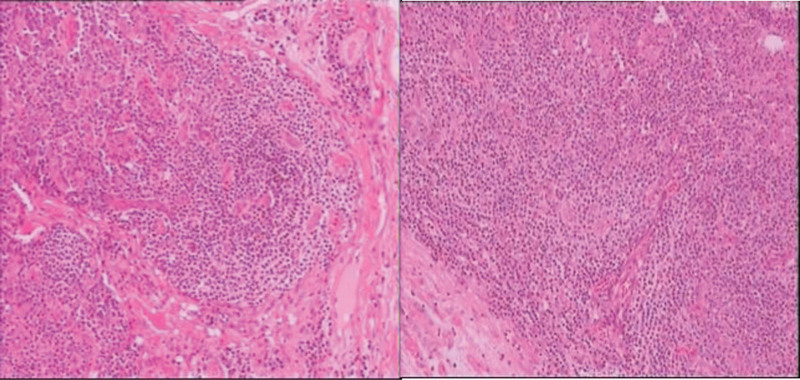
The lymphoid sinuses were dilated, the lymphoid follicles in the cortex exhibited atrophy, and the lymphocytes in the mantle area were arranged in an “onion skin” pattern. A significant number of plasma cells were observed in the interfollicular area and medullary area. Additionally, some plasma cells appeared underdifferentiated, and there was proliferation of small blood vessels.

The diagnosis is: Multicentric Castleman disease (Mixed type) (Fig. 2).

*Treatment*: After confirmation of the diagnosis, the patient received alternating chemotherapy regimens every 6 months, which included cyclophosphamide at 0.6–1 g + VCR at 2 mg + methylprednisolone at 50 mg/5 days, and cyclophosphamide at 1 g + prednisone at 30–50 mg/5 days. This treatment approach was maintained for an average of 6 months per cycle.

The patient has been followed up for 43 months, and at present, her general symptoms have improved compared to before. Additionally, all laboratory indicators, except for platelet count, have mostly returned to normal. Although there is still evidence of abdominal fluid and lymph node enlargement upon reexamination, the condition has improved compared to the past. Therefore, the patient continues to require regular chemotherapy.

*Case 2*: A 58-year-old female patient was admitted with the chief complaint of “discovering a left lower abdominal mass during a health checkup for 10 days.” The patient had noticed a space-occupying lesion in the left lower abdomen during a health checkup at a medical center 10 days ago. She did not experience abdominal distension, abdominal pain, or radiating pain in the waist. There was no fever, and her bowel and bladder habits were normal. Her dietary and sleep patterns were regular, and there had been no significant changes in her recent body weight. She was referred to our hospital with a provisional diagnosis of “left lower abdominal mass.” There was no remarkable medical history, menstrual history, or family history.

Physical examination revealed a palpable mass in the left lower abdomen, approximately 5.0 * 3.0 cm in size, which was tender upon deep palpation and had poor mobility.

**Figure 3.**
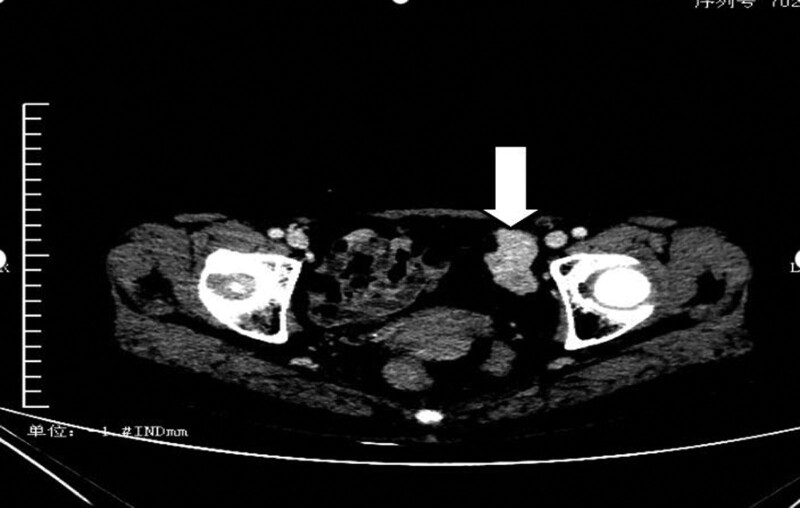
The CT scan indicated a long soft tissue mass near the left iliac blood vessel, which was interpreted as a benign lesion. CT = computed tomography.

Laboratory tests showed elevated ALT at 311 U/L and AST at 200 U/L. Vaginal color Doppler ultrasound indicated a diagnosis of a solid mass near the left iliac vessel. Computed tomography scan showed a long, strip-like soft tissue mass adjacent to the left iliac vessel, with a possibility of benign lesion (Fig. 3). Colonoscopy was negative, and blood routine, tumor markers, and renal function tests were also negative.

**Figure 4.**
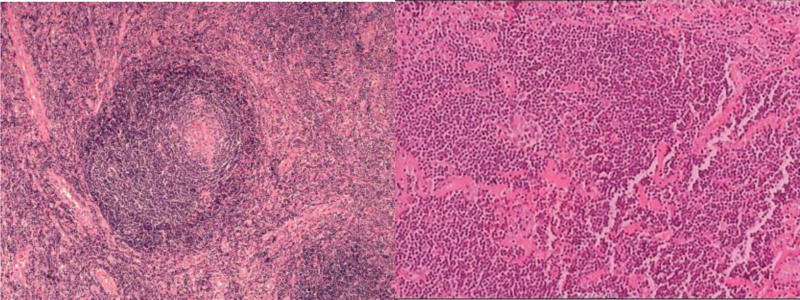
The lymph nodes exhibited an increased number of lymph follicles, with atrophy of the germinal center, and an increase in interfollicular blood vessels. The interfollicular blood vessels inserted vertically into the germinal center, forming a “lollipop” germinal center. This was accompanied by hyalinization of the blood vessel wall and widening of the mantle area, resembling an “onion skin” appearance.

*Treatment*: The patient underwent laparoscopic pelvic mass excision surgery. Intraoperatively, the peritoneum of the pelvic wall was incised along the course of the iliac vessels, revealing a firm mass closely related to the iliac vessels. After careful dissection, the mass was excised. Macroscopically, the specimen was a gray-brown tissue mass, nodular in appearance, measuring approximately 4.5 cm * 2.7 cm * 1.0 cm in size. The margins were clear, and the tissue was firm. Immunohistochemistry results showed CD20(+) in the B area, CD3(+) and CD21 in the T area, CD23 displaying follicular dendritic cell meshwork, CD34 in blood vessels (+), MUM-1 scattered (+), CD68(+), partial IgG(+), Ki-67 in the germinal center at approximately 90%, and around 20% in the rest. The final diagnosis was pelvic retroperitoneal Castleman disease (hyaline vascular type) (Fig. 4). Postoperatively, the patient did not require medication, and there was no evidence of metastasis or recurrence during an 18-month follow-up period.

**Figure 5.**
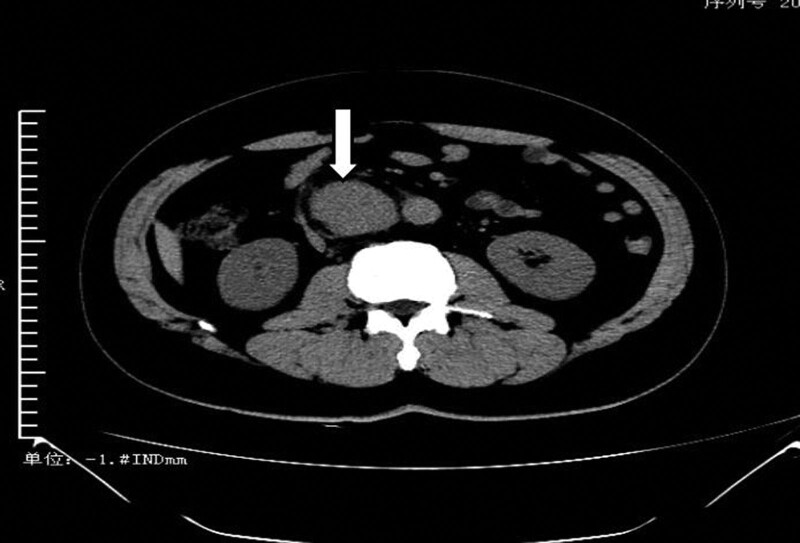
The CT scan revealed multiple nodular soft tissue shadows situated between the abdominal aorta and the inferior vena cava, with the largest measuring 5.0 * 4.0 cm. Additionally, there was compression and deformation of the inferior vena cava. CT = computed tomography.

*Case 3*: A 40-year-old male patient was admitted with the chief complaint of “progressive right upper abdominal and lower back pain for over 6 months.” Six months ago, the patient experienced right upper abdominal and lower back pain without an obvious cause. Initially, the pain was dull and was not taken seriously or treated. However, over time, the pain gradually worsened, changing from a dull ache to a sour and bloating sensation. There were no exacerbating factors, and the use of pain relievers provided slight relief. The patient underwent a computed tomography scan at our hospital, which revealed multiple nodular soft tissue masses between the abdominal aorta and the inferior vena cava, with the largest measuring 5.0 * 4.0 cm. The inferior vena cava was compressed and displaced (Fig. 5).

Physical examination showed a soft abdomen with no tenderness or percussion pain in the lower back. There was no edema in the lower limbs, and laboratory tests did not reveal any abnormalities.

*Treatment*: Surgical intervention was performed, and intraoperatively, the mass was found behind the peritoneum, resembling a pseudomembrane, located at the level of the renal arteries. It enveloped the abdominal aorta and the inferior vena cava on both sides and was closely adjacent to the vertebrae. The tumor had a rich blood supply, and its boundaries were indistinct. After complete dissection from the periphery towards the center, the mass was successfully excised. Macroscopically, the specimen appeared gray-yellow and gray-red, with nodular lesions measuring approximately 5.5 cm * 4.5 cm * 4.5 cm in size. The margins were unclear, and the texture was brittle. Intraoperatively, 1000 mL of blood was lost, and 4 units of blood were transfused.

**Figure 6.**
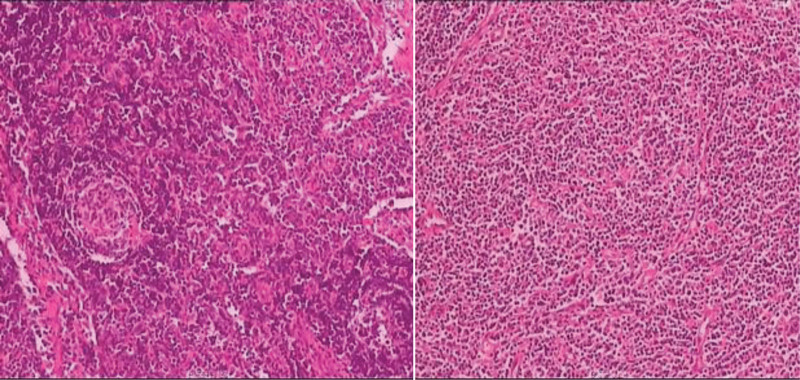
The lymph nodes exhibited follicular hyperplasia, and the interfollicular areas were characterized by a rich presence of plasma cells accompanied by vascular proliferation. Furthermore, there was evidence of degeneration in the lymphoid follicles.

Immunohistochemistry results showed CD3(+), CD15(−), CD20(+), CD30(+), CD21(FDC+), PAX-5(+), IgG4(−), S-100(−), CD68(+), Ki67(70%+), and EBER(−) (Fig. 6).

The final diagnosis was multicentric Castleman disease. The patient did not require medication postoperatively, and during a 21-month follow-up period, there was no evidence of metastasis or recurrence.

## 2. Discussion

CD made its debut in 1956 in a report by Dr Benjamin Castleman on 13 cases of mediastinal lymph node hyperplasia. In this report, Castleman categorized Castleman Disease based on histopathology into hyaline vascular type, plasma cell type, and mixed type.^[8,9]^ Furthermore, based on the involvement of lymph nodes, the disease was categorized into Unicentric Castleman Disease (UCD) and Multicentric Castleman Disease (MCD), ninety percent of the cases documented in the literature were unicentric.^[1]^ The classification is based on the number of involved anatomical sites. According to relevant literature, the incidence of this disease is higher in males and individuals of Caucasian descent, with an average age of diagnosis around 53 years old.^[10]^ The absence of a clear clinical presentation and a definitive definition of the disease makes it challenging to locate high-quality epidemiological literature.

As further research progressed, it was discovered that Castleman disease could occur in any organ, with the majority of cases involving lymphatic organs. However, there were also cases where it affected non-lymphatic organs such as the lungs, spleen, and neck region.^[11]^ This diversity in affected locations contributed to a wide range of clinical presentations.^[12]^

Most studies indicate that patients with the hyaline vascular type tend to exhibit more clinical symptoms, whereas those with the unicentric subtype generally have milder clinical presentations. In this article, among the 3 cases presented, one was classified as the hyaline vascular type, characterized by a single painless mass without significant symptoms. The other 2 cases were diagnosed as multicentric types, and they exhibited complex clinical presentations such as abdominal and lower back pain in case 3, as well as renal impairment, ascites, fever, thrombocytopenia, and fatigue in case 1, which resembled the complex clinical manifestations of TAFRO syndrome.^[12]^

In a recent consensus meeting in Japan, it was proposed to classify multicentric Castleman disease into 2 subtypes based on clinical and histopathological criteria: idiopathic plasma cell type lymphadenopathy and non-idiopathic plasma cell type lymphadenopathy. The latter category encompasses various conditions, including TAFRO syndrome, POEMS syndrome, human immunodeficiency virus-related Castleman disease, lymphoma-associated Castleman disease, and IgG4-related diseases.^[12]^

Among these, TAFRO syndrome, also known as Castleman-Kojima disease, is characterized by systemic inflammatory disorder symptoms, with major features including thrombocytopenia, systemic edema, bone marrow fibrosis, renal dysfunction, and organomegaly. Only a minority of MCD patients exhibit these symptoms, and the majority of thrombocytopenia, anasarca, fever, reticulin fibrosis and organomegaly-idiopathic multicentric Castleman disease-related cases have been reported in Japan. In this multicentric study, the clinical presentations of patients align with the 4 diagnostic criteria of TAFRO syndrome (with negative bone marrow fibrosis). We believe that distinguishing between MCD subtypes can be beneficial for the diagnosis, treatment, and prognosis of this disease.

Treatment for Castleman disease primarily depends on its pathological subtype. Surgical excision of the affected lymph nodes can be considered as the primary treatment for UCD, especially when the diameter of the mass is equal to or >4 cm^[13]^ and it has a favorable prognosis.^[14]^ Laparoscopic surgery can delicately separate the tumor from surrounding tissues, reduce trauma and bleeding, and facilitate postoperative recovery. Based on the advantages of laparoscopic surgery, it can be employed for intra-abdominal CD excision. For patients with a simple presentation, especially those diagnosed with the transparent type, complete surgical removal often leads to a cure in the majority of cases. In situations where surgical excision is not feasible, reducing tumor size can be achieved through radiotherapy and chemotherapy.^[15]^

However, for multicentric cases, especially those diagnosed with the plasmacytic or mixed subtype, clinical presentations are complex, with a tendency toward malignant transformation, and even after treatment, the mortality rate remains high at around 50%, with a survival time of <26 months.^[13]^ In cases where the disease involves multiple sites, surgical excision may not be significantly beneficial. Without prompt treatment, both unicentric and multicentric variants can progress to non-Hodgkin lymphoma.^[16]^

Regarding MCD, there is currently no standardized treatment protocol. Based on relevant literature, treatment options may include chemotherapy regimens similar to those used for lymphoma, such as cyclophosphamide, vincristine (VSR), doxorubicin, and prednisone (the “cyclophosphamide/hydroxydaunorubicin/oncovin/prednisone” regimen), or cyclophosphamide, VSR, doxorubicin, and dexamethasone (the “cyclophosphamide/vincristine/doxorubicin/dexamethasone” regimen). Additionally, radiation therapy and immunomodulators such as steroids, interferon, and thalidomide may offer benefits to MCD patients. In this study, the multicentric Castleman disease patients were treated with alternating chemotherapy regimens consisting of cyclophosphamide, VSR, and prednisone (the “cyclophosphamide/oncovin/prednisone” regimen) and cyclophosphamide plus prednisone, and the patients are still alive, suggesting that the treatment has been effective. However, further follow-up is required to assess the long-term outcomes.

## 3. Conclusion

Due to the lack of specificity in symptoms and affected sites, some clinicians have insufficient awareness of CD, leading to potential misdiagnosis or underdiagnosis. The absence of specific signs in imaging studies and nonspecific blood tests further complicates the diagnostic process. However, tissue biopsy remains a viable option. Therefore, we recommend a thorough examination of suspected CD patients to minimize misdiagnosis and determine the CD subtype for effective targeted treatment.

Surgical excision of affected tissue is the primary method for treating UCD. For patients not suitable for surgery, alternative approaches include radiotherapy and drug therapies such as steroids and immunosuppressive agents. The efficacy of these treatment modalities requires further research. This review aims to provide clinicians with additional clinical guidance, reducing the misdiagnosis rate of CD and offering more effective treatment strategies for affected individuals.

## Author contributions

**Conceptualization:** JinMing Guan, Xin Chen.

**Data curation:** Yang Xu, Shuai Shao.

**Project administration:** JinMing Guan, Xin Chen.

**Writing – original draft:** Yang Xu, Shuai Shao.

**Writing – review & editing:** HaoNan Kang, ZhaoHui Xu, GuoYi Wen, Yan Shan, ZeZhong Gong, Abdulkarem Al-Sharabi, BoXin Qu, YanYing Ren, Fan Zhang.
